# Structural and Functional Roles of Coevolved Sites in Proteins

**DOI:** 10.1371/journal.pone.0008591

**Published:** 2010-01-06

**Authors:** Saikat Chakrabarti, Anna R. Panchenko

**Affiliations:** National Center for Biotechnology Information, National Library of Medicine, National Institutes of Health, Bethesda, Maryland, United States of America; Leeds Institute of Molecular Medicine, United Kingdom

## Abstract

**Background:**

Understanding the residue covariations between multiple positions in protein families is very crucial and can be helpful for designing protein engineering experiments. These simultaneous changes or residue coevolution allow protein to maintain its overall structural-functional integrity while enabling it to acquire specific functional modifications. Despite the significant efforts in the field there is still controversy in terms of the preferable locations of coevolved residues on different regions of protein molecules, the strength of coevolutionary signal and role of coevolution in functional diversification.

**Methodology:**

In this paper we study the scale and nature of residue coevolution in maintaining the overall functionality and structural integrity of proteins. We employed a large scale study to investigate the structural and functional aspects of coevolved residues. We found that the networks representing the coevolutionary residue connections within our dataset are in general of ‘small-world’ type as they have clustering coefficient values higher than random networks and also show smaller mean shortest path lengths similar and/or lower than random and regular networks. We also found that altogether 11% of functionally important sites are coevolved with any other sites. Active sites are found more frequently to coevolve with any other sites (15%) compared to protein (11%) and ligand (9%) binding sites. Metal binding and active sites are also found to be more frequently coevolved with other metal binding and active sites, respectively. Analysis of the coupling between coevolutionary processes and the spatial distribution of coevolved sites reveals that a high fraction of coevolved sites are located close to each other. Moreover, ∼80% of charge compensatory substitutions within coevolved sites are found at very close spatial proximity (< = 5Å), pointing to the possible preservation of salt bridges in evolution.

**Conclusion:**

Our findings show that a noticeable fraction of functionally important sites undergo coevolution and also point towards compensatory substitutions as a probable coevolutionary mechanism within spatially proximal coevolved functional sites.

## Introduction

According to the neutral theory of evolution, the functionality of protein with disadvantageous amino acid substitution can be restored by another amino acid substitution which compensates the first one to sustain the fitness [1]. Such compensatory substitutions together with other factors arising due to common functional, structural and folding constraints lead to covariation between different positions in a protein family [2]. Other positions might not coevolve because they are neutral or under positive selection. Compensatory amino acid substitutions have been described in previous works in terms of their locations in structure, physico-chemical properties [3]–[8] and relation to the diseases [5], [9]. It has been found that interacting residues have tendency to coevolve [4], [5], [10]–[17] and charge compensatory substitutions might make substantial contribution to the residue coevolution [3]–[5], [10], [18]. Although the coevolution is difficult to detect and is rather weak in many cases, the correlated mutations have had comparative success in predicting protein secondary and tertiary structures and in some cases protein interaction partners [19]–[21].

Interestingly, it has been proposed that coupled amino acid changes will mostly occur in the same lineage or on the same branch of the phylogenetic tree [4], [5], [22] due to the strong positive selection pressure to mutate another site to compensate the original mutation. Such lineage specific changes might be important for functional specificity where overall functional constraint remains the same while small tuning of the residue interaction network is required to maintain the new specific functional characteristics. It was shown that residues which form many coevolutionary connections with other residues are more conserved in evolution and are involved in functionally important interactions or conformational changes [17], [23], [24]. It is a subject of extensive study of how coevolutionary processes are related to functional diversification within protein families. Directed evolution experiments, for example, tried to address this question from the practical point of view of designing sequences with certain functional properties by introducing many cumulative compensatory changes [25], [26].

Despite the significant efforts in this field there is still controversy in terms of, the strength of coevolutionary signal, the role of coevolution in functional diversification and affect of structural environment on coevolution. Indeed, coevolution is difficult to detect due to the variable nature of compensatory mutations, the strong dependence of covariations on evolutionary distances, number of sequences in the alignment and residue environment. Moreover, the coevolutionary signal must be separated from the background resulting from various correlations between the non-coevolving residues. The lack of consistency in detecting coevolutionary signal stems from the fact that many methods barring a few [27]–[29] employed so far did not explicitly account for the phylogenetic signal coming from correlations due to phylogenetic relationships between species represented in a given protein family.

Previous studies focused on analyzing coevolution with respect to particular features and processes, for example, disease associated mutations and compensated pathogenic deviations [6], [9], mechanisms of charge compensation [4] and interacting residue coevolution in mammalian proteomes [5]. In this paper we try to fill this gap and study the scale and nature of residue coevolution in maintaining the overall functionality and structural integrity of proteins. Information theory based approaches are widely used to estimate the covariation between sites in protein families [12], [15], [28]–[34]. In the present analysis we use a new, rapid and effective method, MIp, to estimate residue coevolution which is based on information theory and accurately estimates the expected levels of background coming from random and phylogenetic signals [29]. It has been shown, for example, that MIp can identify higher number of contacting residues compared to other coevolution detecting methods [29]. We employed a large scale dataset of protein families extracted from a well curated Conserved Domain Database (CDD) [35] to study the evolutionary, structural and functional aspects of coevolved residues.

We found that the networks representing the coevolutionary residue connections within our dataset are in general of ‘small-world’ type; they have clustering coefficient values higher than random networks and also show much lower mean shortest path values compared to random and regular networks. We also found that altogether 11% of functionally important sites are coevolved with any other sites. Active sites are found more frequently to coevolve with any other sites (15%) compared to protein (11%) and ligand (9%) binding sites. Metal binding and active sites are also found to be more frequently coevolved with other metal binding and active sites, respectively. Supporting the previous findings [15], [16], [28], [29] our analysis of the coupling between coevolutionary processes and the spatial distribution of coevolved sites also shows that a high fraction of coevolved sites are located close to each other. Moreover, ∼80% of charge compensatory substitutions are found at very close spatial proximity (< = 5Å), pointing to the possible preservation of salt bridges in evolution.

## Results

### Coevolutionary network of protein sites

**Figure 1:**
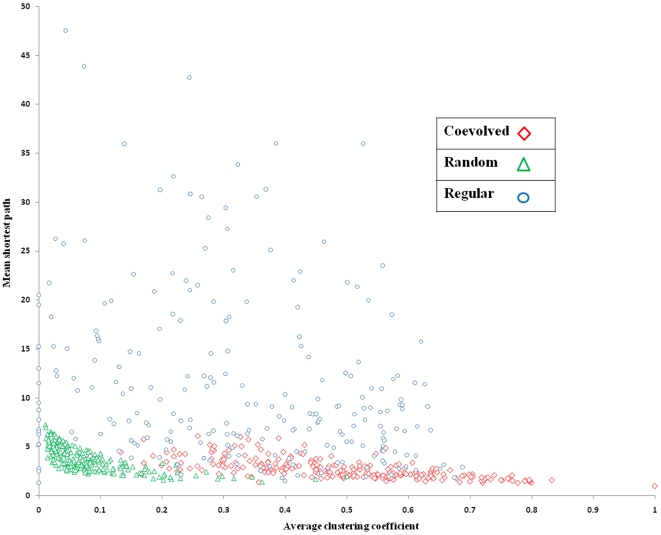
‘Small-world’ characteristics of coevolved networks. Shortest paths and clustering coefficients are calculated and plotted for each family/network that has average degree (k) equal or more than 2.

Altogether we identified 39527 coevolved site pairs from 803 family alignments. Figure 1 shows the mean shortest path lengths plotted versus the average clustering coefficients of the coevolved networks for each family (red diamonds) and for the corresponding random and regular networks generated from equivalent number of nodes and edges in each family [36]. Table 1 shows the means and standard errors of clustering coefficients and shortest path lengths calculated by averaging over all 244 families/networks with average degree (k) equal or more than 2. It is clear from Figure 1 and Table 1 that unlike random and regular networks, coevolutionary networks have high clustering coefficients and low mean shortest path lengths. Random networks are characterized by low clustering coefficients and small shortest path lengths while regular networks usually have larger clustering coefficients and high shortest path lengths [36]–[40]. Hence, we can conclude that the protein coevolutionary networks, in general, are of a ‘small-world’ type. Small-world network is a type of ‘graph’ in which most nodes (in our case coevolved protein sites) are not neighbors of one another, but most nodes can be reached from every other by a small number of steps [36]–[40].

**Table 1:** Network properties.

Network	Mean clustering coefficient	Mean shortest path
**Coevolved**	0.49 (±0.01)	2.74 (±0.07)
**Random**	0.09 (±0.00)	3.78 (±0.08)
**Regular**	0.32 (±0.01)	11.30 (±0.56)

### Functional sites and their coevolutionary networks

**Figure 2:**
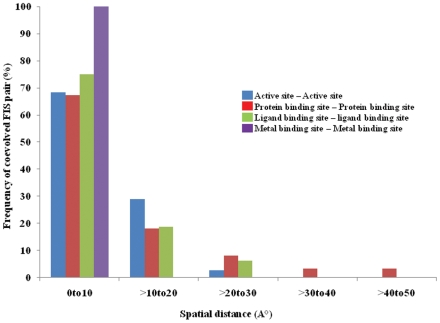
Spatial distribution of coevolved functionally important sites. Frequencies of coevolved functionally important site pairs are plotted versus the spatial distances between them. Bars represent frequencies of coevolved connection within each functional category.

To examine the coevolution of functionally important sites (FIS), we calculated the fraction of coevolved sites that are involved in important molecular functions such as catalysis, protein, ligand or metal binding, and post translational modifications (Tables 2 and 3). We found that altogether 11% of FIS (430 out of 3989) are coevolved with any other sites. Active sites are found more frequently to coevolve with any other sites (15%) compared to protein (11%) and ligand (9%) binding sites (Table 2). Metal binding and active sites are also found to be more frequently coevolved with other metal binding and active sites, respectively (Table 3). Close examination of these coevolved functional sites reveals that vast majority of coevolved FIS are located at relatively small distances of less than 10Å (Figure 2).

**Table 2:** Coevolution of functionally important sites (FIS).

Number of families	Category of sites	Number of FIS	Number of all coevolved site pairs	Category of sites	Number of coevolved FIS
197	All	3989	5405	All	430 (11%)
	Active	795		Active	116 (15%)
	Protein binding	1498		Protein binding	169 (11%)
	Ligand binding	1369		Ligand binding	118 (9%)
	Metal binding	171		Metal binding	14 (8%)
	Post-translational modification	43		Post-translational modification	3 (7%)
	Miscellaneous	113		Miscellaneous	10 (9%)

**Table 3:** Functional coupling of coevolved sites.

FIS	Total coevolved pairs	% of coevolved site pairs						
		Active site	Protein binding site	Ligand binding site	Metal binding site	Post-translational modification site	Miscellaneous site	Non Functional site
**Active site**	345	22.00	0.87	0.29	0.58	0.00	0.58	76.00
**Protein binding site**	285	1.05	21.40	1.05	0.00	0.00	0.35	76.14
**Ligand binding site**	189	0.53	1.59	8.47	0.53	0.00	0.00	88.89
**Metal binding site**	42	4.76	0.00	2.38	33.33	0.00	0.00	59.52
**Post-translational modification site**	3	0.00	0.00	0.00	0.00	0.00	0.00	100.00
**Miscellaneous site**	12	16.67	8.33	0.00	0.00	0.00	8.33	66.67

**Figure 3:**
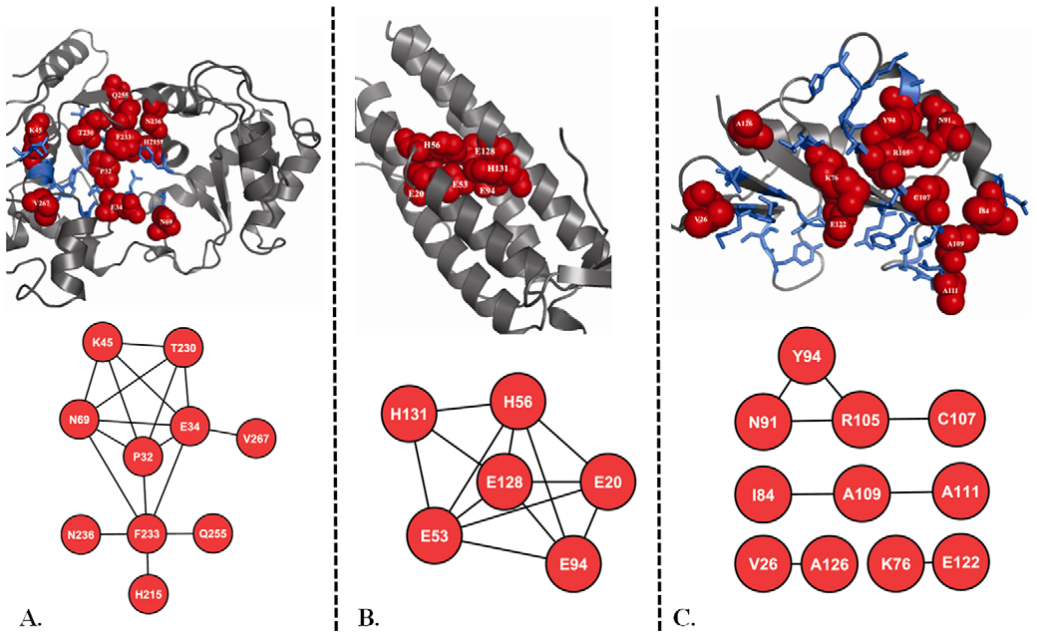
Examples of coevolved functional sites. Coevolved (panel A, marked in red spheres) and non-coevolved (panel A, marked in blue sticks) active sites are projected onto the structure of a representative member (PDB code: 1EUQ) from Glutamyl-tRNA synthetase(GluRS)/Glutaminyl-tRNA synthetase (GlnRS) catalytic domain family (CDD code: CD00418). Here an edge connects two coevolved residues (red circles). Coevolved metal binding sites (panel B, marked in red spheres) are projected onto the 3D structure of a representative member (PDB code: 1LKO) from ferritin-like diiron-carboxylate protein domain family (CDD code: CD00657). Coevolved (panel C, marked in red spheres) and non-coevolved (panel C, marked in blue sticks) protein binding sites are projected onto the structure of a representative member (PDB code: 1JD1) from YjgF_YER057c_UK114_family (CDD code: CD00448). Protein structural and network representations were created using the PyMol [47] and Cytoscape [48] program.

After examining the network properties of functionally important coevolved sites we found that overall functional sites have lower tendency to form coevolutionary clusters compared to all coevolved sites as suggested by their lower average clustering coefficients values (0.19 compared to 0.49 for all coevolved sites, Table 4). Within the functional sites, metal binding sites have the highest tendency to form coevolutionary clusters while ligand binding sites have lowest tendency (the difference is statistically significant, t-test p-value is less than 0.02). Active and protein binding sites have intermediate propensity towards forming coevolutionary clusters (Table 4). Figure 3 provides examples of highly connected coevolved metal binding sites from ferritin-like diiron-carboxylate protein domain family, CDD code: CD00657) and moderately connected coevolved active and protein binding sites from Glutamyl-tRNA synthetase(GluRS)/Glutaminyl-tRNA synthetase (CDD code: CD00418) and YjgF_YER057c_UK114_family (CDD code: CD00448), respectively.

**Table 4:** Network properties of coevolved sites.

Sites	Average clustering coefficient	Mean shortest path	Average degree
All sites	0.49(±0.01)	2.74(±0.07)	3.31(±0.04)
All functional sites	0.19(±0.04)	1.41(±0.04)	1.55(±0.08)
Active sites	0.12(±0.05)	1.51(±0.06)	1.75(±0.17)
Ligand binding sites	0.05(±0.04)	1.32(±0.06)	1.29(±0.05)
Protein binding sites	0.18(±0.06)	1.39(±0.06)	1.44(±0.09)
Metal binding sites	0.32(±0.19)	1.41(±0.12)	2.00(±0.45)

### Structural features of coevolved sites

**Figure 4:**
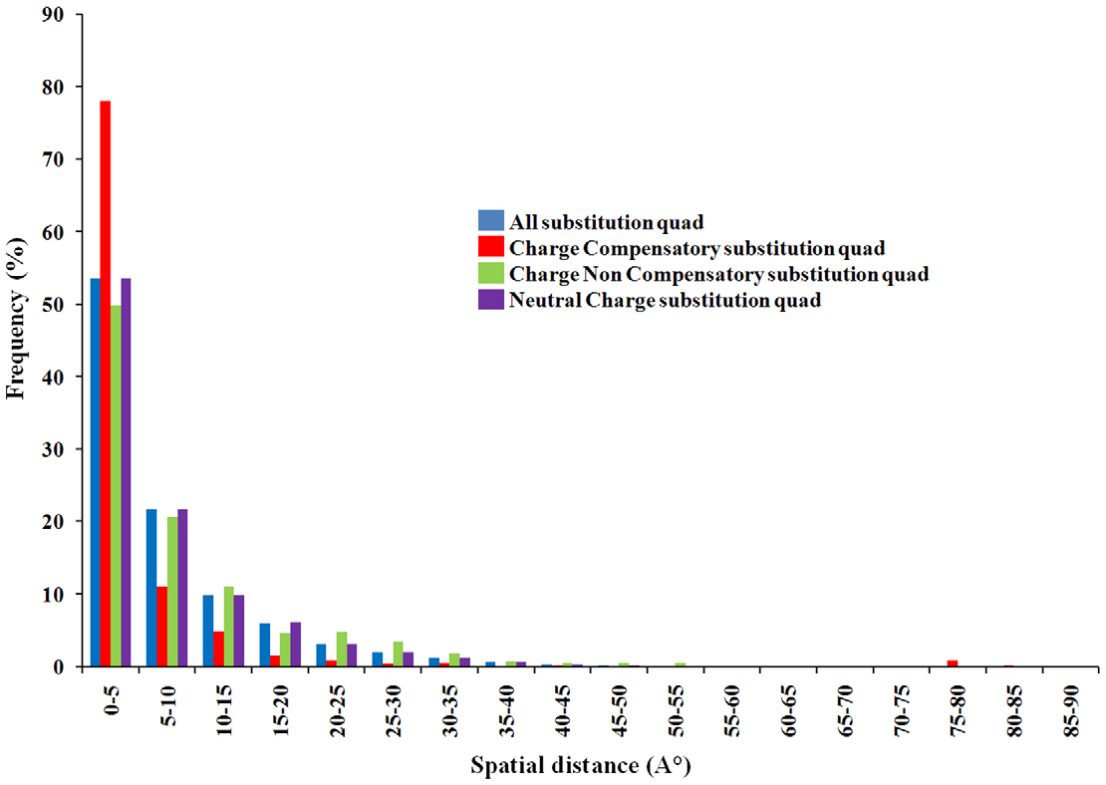
Charge compensatory substitutions within the coevolved sites. Frequency of charge compensatory substitutions was calculated by counting the number of charge compensatory substitution quads (see Methods for details) for each pair of coevolved alignment columns. Frequency of charge compensatory substitutions (Y axis) is plotted against the spatial distances (X axis) between coevolved residue pairs.

We addressed the question of how a pair of coupled residues evolves and manifests in compensatory substitutions which are typically apparent from the analysis of spatial distances between coevolved sites. We observed that a high fraction of coevolved sites prefers spatial proximity, namely 53% of coevolved pairs are within 10Å distance from each other while 80% resides within 20Å distance from each other (shown by blue bars in Figure 4). We also compared distance distributions for the datasets of less and more than 125 sequences (see Methods) with the distance distribution of randomly selected non-coevolved site pairs. We found that there is a statistically significant difference (p-value≪0.01) between mean spatial distances of the non-coevolved sites and coevolved sites from these datasets (Figure S1 and Table S1). To decipher the specific mechanisms of such distance dependence we analyzed the amino acid content of coevolved site pairs at the substitution quads (see Methods) extracted from each pair of protein sequences within the multiple sequence alignment. We especially focused on charge compensatory quads where the fitness of opposite charge interactions is generally preserved by compensating the impact of substitutions at the interacting residues during the course of evolution. Charge compensatory substitutions among the coevolved sites were investigated by calculating the frequency of charge compensatory substitution quads for each pair of coevolved alignment columns. Figure 4 shows the fraction of various charge substitution quads within the coevolved sites with respect to their spatial distances. We observed that ∼80% of charge compensatory substitutions are found at very close spatial proximity of less than 5Å. It indicates that these substitutions might be maintained in evolution to preserve the salt bridges. Similarly, we also found that the spatial distance distribution of charge compensatory substitutions is significantly different (p-value≪0.01) than that of non-coevolved columns pairs (Figure S2).

To detect subtle stereo-chemical variations which lead to coevolution of sites, we compared physico-chemical properties of amino acids at each coevolved site pairs. Correlation coefficients of physico-chemical properties of residues between two coevolved sites/aligned columns were calculated. The higher the correlation coefficient, the larger is the similarity of physico-chemical properties between two coevolves sites. The histogram of values of correlation coefficients is presented in Figure S3. As can be seen from this figure, coevolved sites have more similar physico-chemical properties compared non-coevolved sites. Systematic categorization of coevolved residue pairs into conservative and non-conservative types (see Methods for definitions) also reveals that coevolved pairs formed by similar volume amino acids (conservative and neutral coupling) are more prevalent (Figure S4) than non-conservative coupling (parings of amino acids that have large difference in volume). Opposite charged residues have a higher preference to coevolve although charge-neutral couplings (charge: non-charge pairings) are most prevalent in coevolved residue pairs (Figure S4). This is consistent with our previous observation on charge compensatory quads that oppositely charged residues can be preferred more at closer distances probably to maintain the salt bridges.

**Figure 5:**
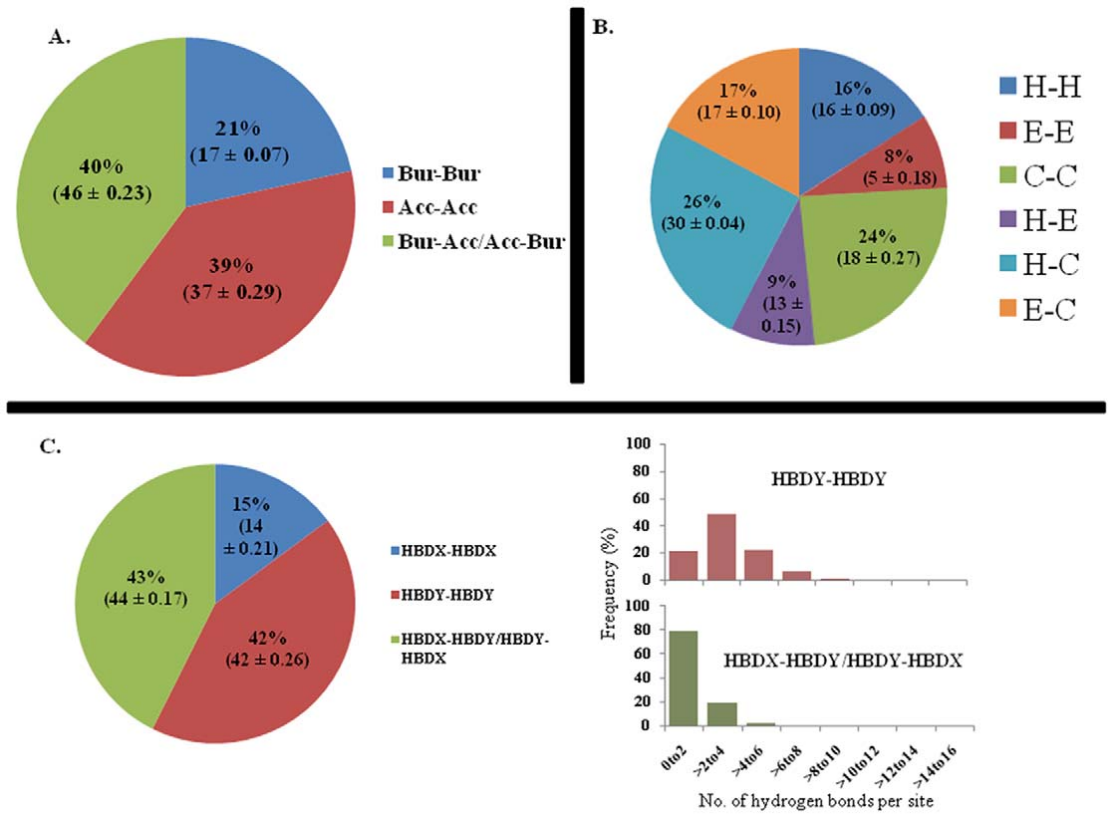
Percentage of sites with given structural properties is shown. Structural properties such as solvent accessibility (panel A), type of secondary structures (panel B) and hydrogen bonds (panel C) were calculated for coevolved sites. Solvent accessibility (Buried: Bur; Accessible: Acc) was measured using the PSA program from JOY package [44]. Within the coevolved site pairs, if both residues are buried or accessible, they are shown as ‘Bur-Bur’ or ‘Acc-Acc’, respectively. Secondary structure [helix (H), strand (E) and coil (C)] distribution for coevolved residue pairs is shown in panel B. Hydrogen bonding patterns were estimated using the HBOND programs from the JOY package. ‘HBDY-HBDY’ and ‘HBDX-HBDX’ indicate cases where both coevolved residues are involved or not involved in hydrogen bonding correspondingly. ‘HBDX-HBDY/HBDY-HBDX’ indicates cases where at least one residue is involved in hydrogen bonding. Values in the parenthesis show mean and standard error of estimated from the distribution of structural property values for randomly selected non-coevolved residue pairs (5 randomizations were performed).

We also examined different structural properties such as solvent accessibility, secondary structures, and hydrogen bonding patterns of coevolved sites (Figure 5). We found that there is a statistically significant bias of coevolved sites to be either both buried or both accessible compared to non-coevolved sites (chi-square contingency test p-value≪0.01). At the same time we observed a certain tendency of both coevolved sites to be both in the coil secondary structure assignments (p-value≪0.01). Similar patterns are observed for the datasets with less and more than 125 sequences in the alignments (Figure S5).

### Examples of coevolved sites

**Figure 6:**
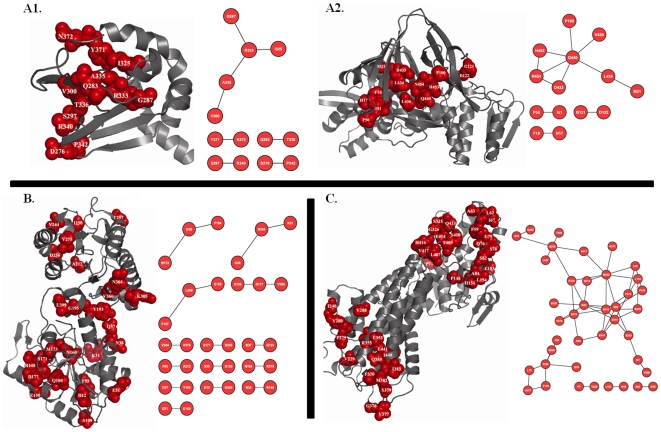
Examples of coevolved sites. Panel A shows two examples where coevolved sites of CAP family of transcription factors (CDD code: cd00038; PDB code: 1RGS) and hedgehog/intein domains (CDD code: cd00081; PDB code: 1DQ3) are projected on their representative protein structures. Panel B shows an example of phosphoglycerate kinase family (CDD code: cd00318; PDB code: 1QPG) while panel C shows an example of coevolutionary connections from phenylalanine ammonia-lyase (PAL) and histidine ammonia-lyase (HAL) domain family (CDD code: cd00332; PDB code: 1GK2). Network representations were created using PyMol [47] and Cytoscape [48] program.

Figure 6 provides examples of coevolved sites projected on the 3D structures of the representative members of protein families from our dataset. Figure 6A shows two examples where coevolved sites of CAP family of transcription factors (CDD code: CD00038; PDB code: 1RGS) and hedgehog/intein domains (CDD code: CD00081; PDB code: 1DQ3) are projected on their representative protein structures, respectively. Coevolutionary network is shown where nodes represent the coevolved sites while an edge is drawn between two sites which coevolve. In these two examples, none of the coevolved site pairs are located more than 10Å apart from each other. Similarly, Figure 6B shows an example of phosphoglycerate kinase family (CDD code: CD00318; PDB code: 1QPG) where a significant fraction (36%) of the coevolved sites are located more than 20Å and less than 40Å apart from each other. Figure 6C shows an example of coevolutionary connections from phenylalanine ammonia-lyase (PAL) and histidine ammonia-lyase (HAL) domain family (CDD code: cd00332; PDB code: 1GK2) where coevolved sites are located far away (>40Å) from each other.

## Discussion

The new mass of evidence points to the importance of coevolution in shaping the protein function. Protein function is determined by interactions with other cell components and by residue-residue interactions. Residue interactions important for the protein functional integrity are conserved in evolution. At the same time proteins change their function in evolution and therefore some functional sites are under positive selection to change in order to accommodate new functional specificities. Certain variability coupled together with the strong functional constraints and the involvement in the network of interactions makes functional sites an ideal target for coevolutionary processes. Indeed, it has been shown previously that many coevolved positions are located at or near functionally important sites [16], [24] and pathogenic missense mutations can be compensated by another mutation to restore the fitness [6], [9]. Moreover, recently we showed that coevolutionary processes are directly related to functional diversification within protein families and the sites determining functional specificity often coevolve [17].

In accordance with the hypothesis outlined in the previous paragraph that noticeable number of functionally important sites might undergo coevolution, we observe that 15% of all active sites coevolve with any other sites and 22% of these coevolutionary relationships include pairs between two active sites. Protein and ligand binding sites are shown to coevolve with other sites in about 11% and 9% of the cases and coevolve exclusively with each other (protein binding sites coevolve with protein binding sites and ligand binding site coevolve with ligand binding site) in 21% and 9% of the cases respectively. After examining the properties of coevolutionary networks, we found that it has “small-world” properties and is characterized by high clustering coefficient and low mean shortest path length. Furthermore, we found that on average sites coevolve with about three other sites while functional sites in particular coevolve with about 1.5 sites. These findings point to multiple coevolutionary events between multiple residues in proteins which make it different from the coevolution between, for example, hydrogen bonded base pairs in RNA [16]. In addition, coevolved functional sites with the exception of metal binding sites form less dense and more disjoint (low clustering coefficient with low mean shortest path) networks compared to all coevolved sites. The less dense network can be explained by the presence of multiple patches and clusters of spatially separated binding and active sites. On the contrary, metal binding sites form quite dense coevolutionary networks which are consistent with their tendency to form a lot of hydrogen bonding, stacking and hydrophobic interactions between each other to provide the ion coordination and contribute to both stability and functionality of the protein.

To decipher the mechanisms of coevolution between functional sites we looked whether their coevolution was coupled with the spatial proximity and found that this is indeed the case. Vast majority of coevolved functional sites are within close to proximity to each other, especially metal binding and active site residues. This observation points to the compensatory substitutions as a probable coevolutionary mechanism within these spatially proximal functional sites. Interestingly, more than 20% of protein binding sites exhibit long distance coevolution between each other (on distances larger than 20 Å) which might be caused by allostery, intramolecular dynamics or common constraints imposed by the binding partner. We also performed an analysis of all coevolved ion pairs in our dataset and it was not surprising to see charge compensations and their dependence on the spatial distance. What was surprising is to see that almost 80% of charge compensatory quads are located at very short distance from each other of less than 5Å (this is not the case for non-charge compensatory quads). This observation implies a strong compensatory component in the coevolution between charged residues forming salt bridges. The strong tendency of correlated ionic interactions to be spatially coupled has been observed previously in the study involving the double replacements of interacting positions (DRIP) [5]. In our work we addressed this question from a completely different angle. We first identified the coevolved sites/quads on the large scale set of protein domains using the state of art coevolutionary detection methods and subsequently analyzed them in terms of the distances and physico-chemical properties. Even though the fraction of charge compensatory substitutions among all coevolved pairs is rather small, we argue that they might make an important contribution in the functional diversification as electrostatic interactions play essential role in specific binding and residue interactions.

## Methods

### Dataset

We collected protein domain alignments from version 2.13 of the Conserved Domain Database (CDD), the most current version of which is available at http://www.ncbi.nlm.nih.gov/Structure/cdd/cdd.shtml. CDD multiple alignments have been manually curated to reconcile sequence alignments with protein three-dimensional structures and structure-structure superposition [35]. 803 CDD domain alignments were selected which have at least one structure entry and more than 25 domain family members. We also excluded small CDD families with alignments of less than 50 residues long. A list of CDD domain families used in this study is provided in Table S2.

Previous studies [27]–[29], [32] suggested that ‘random MI’ signal might arise if the input alignments do not contain enough number of sequences which may in turn lead to false predictions of coevolved sites. To verify whether the main conclusions in the paper hold true for the families with smaller number of sequences we generated the datasets with less than 125 sequences in the alignments (622 alignments) and the dataset with more than 125 sequences in the alignments (181 alignments). We compared the distance distributions and structural properties for these datasets as well as whole dataset with the distributions of randomly selected non-coevolved site pairs. Random non-coevolved sites were identified using 1000 independent randomization cycles. It should be mentioned that the number of families with experimentally annotated functionally important sites (FIS) is quite limited; in our study we used 197 CDD families with at least one known FIS and at least one pair of coevolved sites. We found that in this dataset only 11% of FIS (430 out of 3989) are coevolved with any other sites. Filtering out the CDD families with less than 125 sequences drastically reduces the number of coevolved site pairs and coevolved FIS per family and makes the analysis of family coevolutionary networks impractical.

### Identification of coevolved sites

Mutual Information is a widely used measure to estimate the covariation between sites in protein families. However, its usefulness has been limited by factors like its relative inability to handle positions with higher entropy, alignments with lower number of sequences and filtering the background phylogenetic signal arising due to the phylogenetic relationships between the organisms represented in the family [12], [27]–[29], [32], [41].

In this analysis we used a rapid and effective method to estimate coevolutionary connection between two sites of a protein family [29]. This method (MIp) is based on information theory that accurately estimates the expected levels of background coming from random and phylogenetic signals. Removal of the phylogenetic and random background noise allows to identify substantially more significant coevolving positions in protein families and it has been shown that MIp can identify higher number of contacting residues compared to other methods of detecting coevolution [29]. Altogether we identified 39527 coevolved site pairs from the 803 family alignments with MIp Z-score cutoff of 4.0 or higher.

### Calculation of network parameters

We analyzed the properties of networks for each family where nodes were represented by residue sites and edges by coevolutionary connections between them.

The clustering coefficient *C_i_* for a position (node) *i* in the coevolutionary graph is calculated as the ratio of the number of edges between immediate neighbors of node *i* and the maximum possible number of edges which could exist between the neighbors.
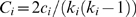
where *k_i_* is the degree of node *i*. The mean clustering coefficient was calculated by averaging the clustering coefficients (*C_i_*) for all nodes in the network. The mean shortest path length *L_coev_* for a coevolved network was calculated using Johnson's algorithm [42] as the average of shortest paths among all unique pairs of nodes. The average clustering coefficients and mean shortest path lengths for random and regular networks were calculated according to the following formulae [36], [37], [39]

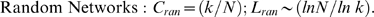


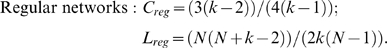
where N is the number of nodes and *k* is the number of edges. For the network analysis, we excluded families for which average degree (k) is less than 2 so that C*_reg_* could be defined and ended up with 244 families.

### Different categories of coevolved sites

A total of 3989 functional sites were extracted from 197 CDD multiple alignments (subset of the 803 alignment dataset which had functional site annotations; Table S3) that have been categorized into six functional categories using protein structures, literature, and experimental data annotations available for each CDD domain [35], [43]. These sites cover a broad range of molecular functional categories, including 795 active sites, 1369 ligand binding sites, 1498 protein–protein binding sites, 171 metal binding sites, 41 post-translational modification sites, and 113 sites with miscellaneous functions.

To analyze how the change in one protein site is compensated in evolution by changing another site in a homologous protein, we used compensatory substitution quads. Each quad represents instances of simultaneous variations in two positions from two sequences and can be illustrated on the example of charge compensatory substitutions. Charge compensatory substitution quad can be explained using the following example.
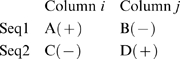



A and D are positively charged while B and C are negatively charged residues. A charge compensatory substitution can be seen when the overall charge is maintained by compensating the impact of mutation of A to C (positive to negative charge) by the simultaneous mutation of B to D (negative to positive charge). This evolutionary phenomenon is called charge compensatory substitution. Similarly, in charge non-compensatory substitution quad, four charged residues do not get coupled in a compensatory manner. All other substitution quads involving either charged or non-charged residues were categorized as “neutral”. Occurrence of charge compensatory substitutions was investigated by calculating the frequency of charge compensatory substitution quads for each pair of coevolved alignment columns.

### Structural and physico-chemical properties of coevolved sites

Spatial distances between two protein residues were calculated utilizing the nearest protein atom coordinates supplied in the individual PDB [44] file. Structural properties such as solvent accessibility, secondary structures, and hydrogen bonds were computed from the protein structure using the JOY package [45]. Solvent accessibility was measured using the PSA program from JOY package and residues that have accessible surface area less than 7% were treated as solvent buried or inaccessible. Similarly, secondary structures (helix, strand and coil) and hydrogen bonding patterns were estimated using the SSTRUC and HBOND programs from the JOY package, respectively. Physico-chemical properties (such as hydrophobicity, polarity, charge etc) were obtained from the UMBC AAIndex database [46] (Table S4) and were utilized to distinguish similarity between two coevolved sites.

All amino acids were also categorized into two groups based on their volume and charge following the scheme described previously [3]. Covariations between residues which differ from each other by not more than volume of a methyl group (∼30Å) are called ‘conservative coupling’ whereas residue pairs with volume difference of more than one or two methyl groups are categorized as neutral coupling. Larger volume deviations are marked as ‘non-conservative coupling’ (Table S5). Similarly, pairing between two oppositely charged residues is termed as ‘non-conservative’ while a ‘conservative coupling’ is constituted by two similarly charged residues. Likewise, coevolved residue pairs where one residue is charged while the other is not are termed as ‘neutral coupling’.

## Supporting Information

Figure S1 Spatial distribution of coevolved and non-coevolved sites. Frequencies of coevolved and non-coevolved site pairs are plotted versus the spatial distances between them. Distance distribution of coevolved sites from the whole dataset (803 alignments; panel a), <125Seq dataset (622 alignments; panel b) and > = 125Seq datasets (181 alignments; panel c) is compared against randomly selected non-coevolved site pairs.
(0.07 MB DOC)

Figure S2 Frequency of charge compensatory substitutions (Y axis) of coevolved and non-coevolved sites are plotted against the spatial distances (X axis) between coevolved and non-coevolved residue pairs, respectively.
(0.05 MB DOC)

Figure S3 Similarity in physico-chemical properties within coevolved residues. Correlation coefficients (X axis) were calculated between two coevolved sites utilizing matrices (values normalized from 0 to 1) of 13 non redundant physico-chemical properties (such as hydrophobicity, polarity, charge etc) obtained from the UMBC AAIndex database. Non-coevolved pairs were selected by randomly picking two sites from a pool of non-coevolving sites within each protein family. Histogram corresponding to the coevolved sites is shifted toward larger positive values compared to the histogram of correlation coefficients calculated for randomly selected non-coevolved sites (p-value<10−4) indicating that coevolved sites have more similar physico-chemical properties compared non-coevolved sites.
(0.10 MB DOC)

Figure S4 Conservative and non-conservative coevolved residue pairs. Residue pairs were categorized according to volume (a) and charge (b) of amino acids. Covariations between residue pairs differing not more than volume of a methyl group (∼30Å) are termed as ‘conservative coupling’ where residue pairs with volume difference of more than one or two methyl groups are categorized as neutral coupling. Larger volume deviations are marked as ‘non-conservative coupling’ (please see Table S5 for details). Similarly, pairing between two oppositely charged residues is termed as ‘non-conservative’ while charge a ‘conservative coupling’ is constituted by two similarly charged residues. Coevolved residue pairs where one residue is charged while the other is not are termed as ‘neutral coupling’. Frequencies (normalized by the number of all coevolved residue pairs) of conservative, non-conservative, and neutral residue pairs were plotted with respect to the spatial distances (X axis) between the coevolved residue pairs.
(0.26 MB DOC)

Figure S5 Structural properties such as solvent accessibility (panel a), type of secondary structures (panel b) and hydrogen bonds (panel c) for the coevolved sites were compared with that of randomly selected non-coevolved site pairs.
(0.18 MB DOC)

Table S1 Mean distances for randomly selected non-coevolved site pairs are provided within parenthesis. Significance of the test was determined with respect to the mean distances of randomly selected non-coevolved pairs (1000 randomization cycles).
(0.03 MB DOC)

Table S2 (0.03 MB TXT)

Table S3 (0.01 MB TXT)

Table S4 (0.03 MB DOC)

Table S5 (0.03 MB DOC)
