# Supplementary figures and images for: Structural and Functional Roles of Coevolved Sites in Proteins
Source: PLoS One. 2010 Jan 6;5(1):e8591. doi: 10.1371/journal.pone.0008591 (PMC2797611; doi:10.1371/journal.pone.0008591)

Supporting Information File 1:

Authors: Saikat Chakrabarti and Anna R. Panchenko

**Figure S1.**

**
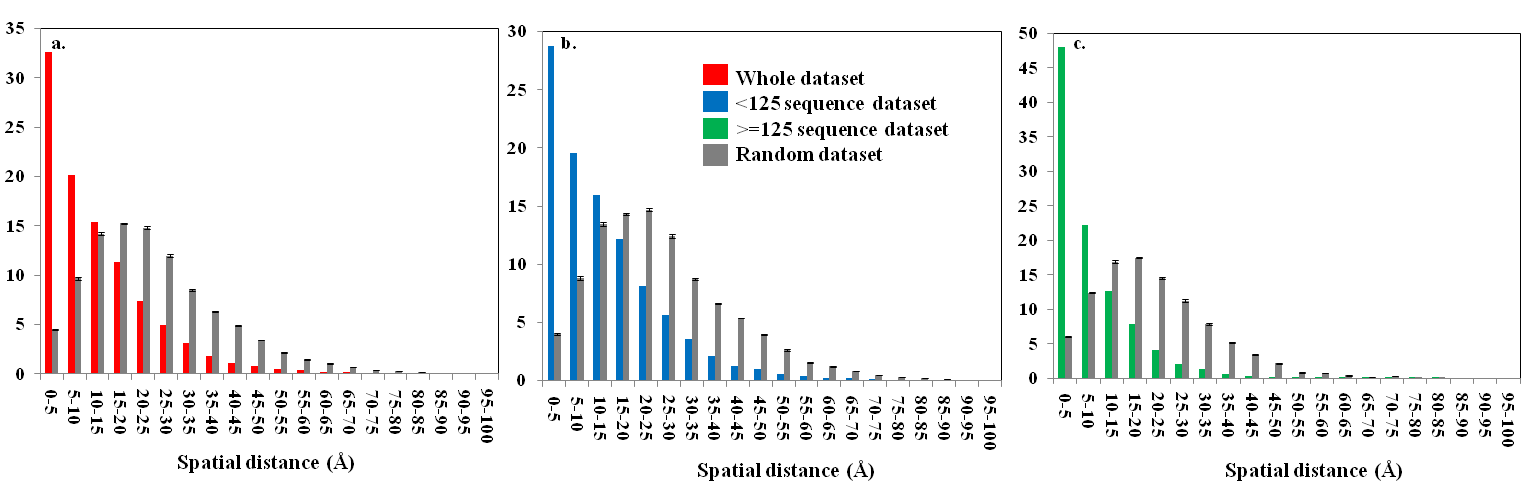
**

Supplement: Figure S1 — Spatial distribution of coevolved and non-coevolved sites. Frequencies of coevolved and non-coevolved site pairs are plotted versus the spatial distances between them. Distance distribution of coevolved sites from the whole dataset (803 alignments; panel a), <125Seq dataset (622 alignments; panel b) and > = 125Seq datasets (181 alignments; panel c) is compared against randomly selected non-coevolved site pairs. (0.07 MB DOC) [file pone.0008591.s001.doc]

Supporting Information File 3:

Authors: Saikat Chakrabarti and Anna R. Panchenko

**Figure S2.**

**
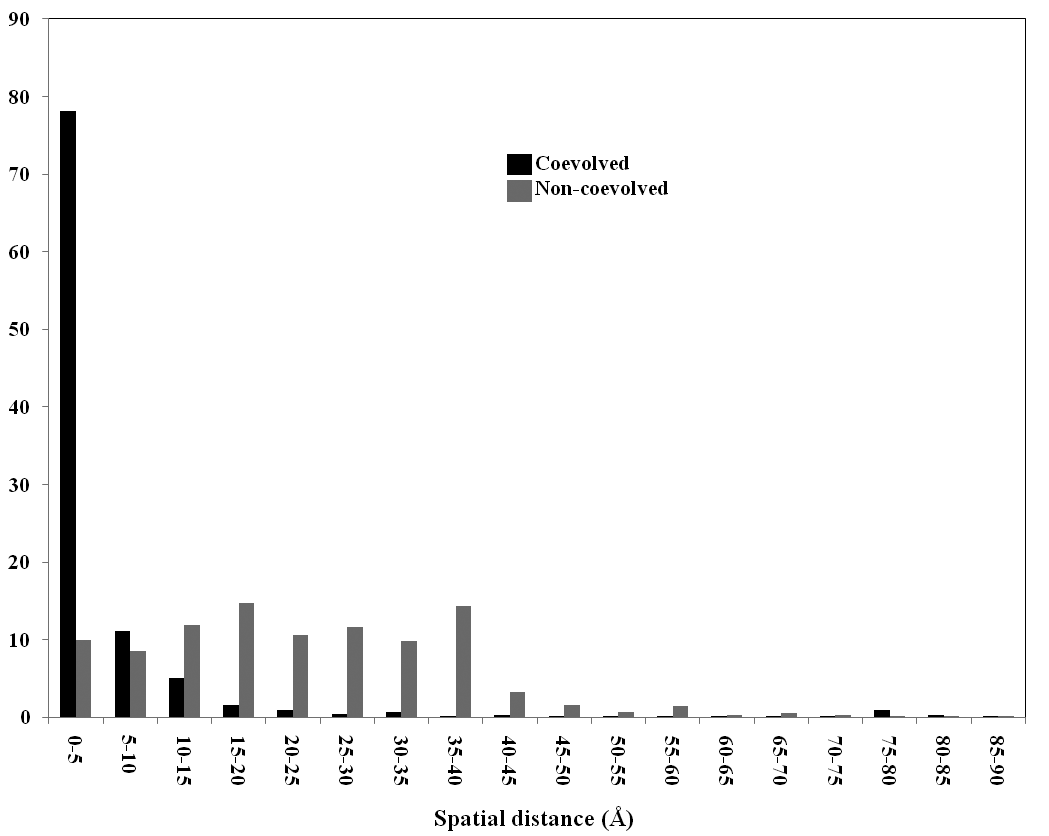
**

Supplement: Figure S2 — Frequency of charge compensatory substitutions (Y axis) of coevolved and non-coevolved sites are plotted against the spatial distances (X axis) between coevolved and non-coevolved residue pairs, respectively. (0.05 MB DOC) [file pone.0008591.s002.doc]

Supporting Information File 4:

Authors: Saikat Chakrabarti and Anna R. Panchenko

**Figure S3**


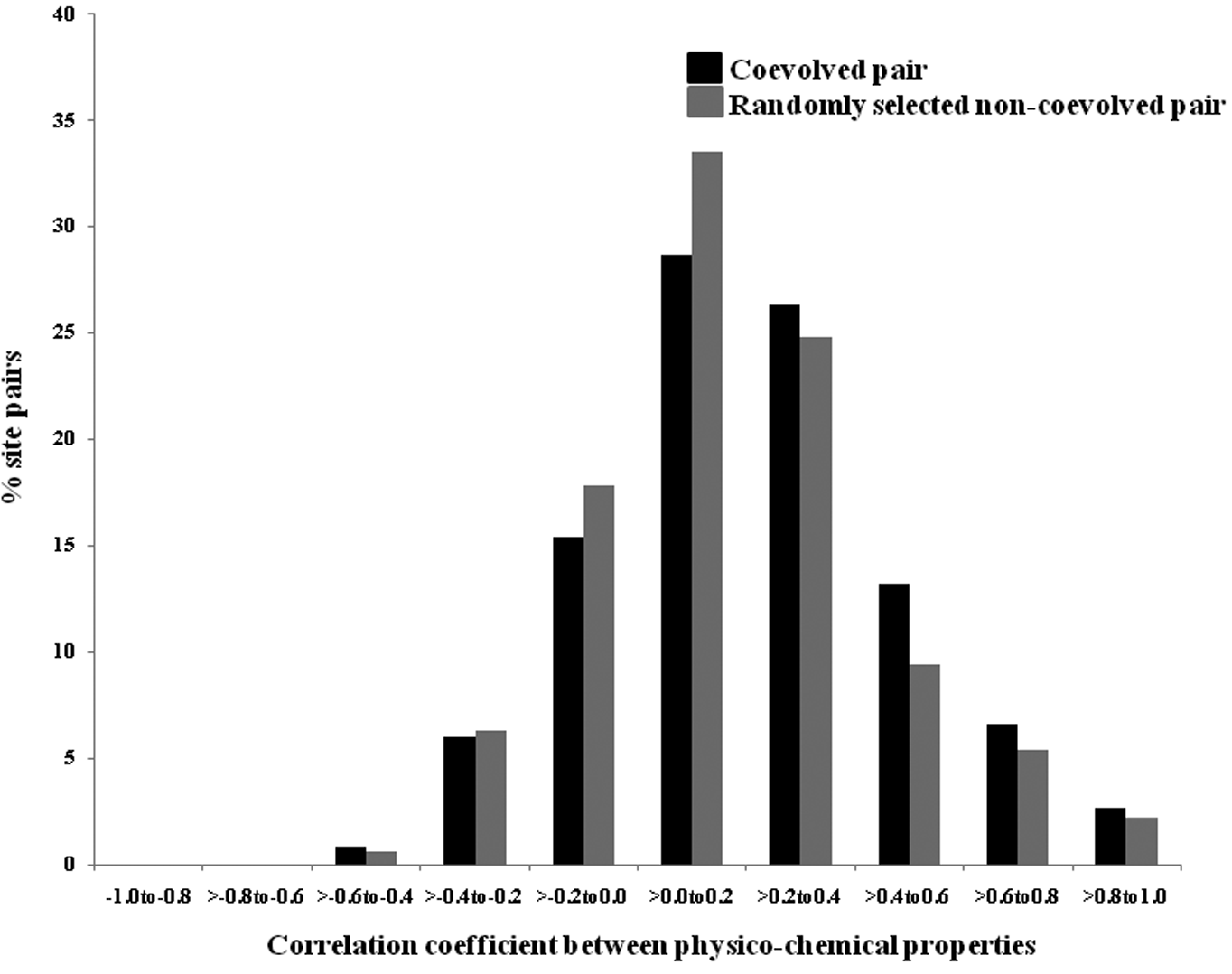

Supplement: Figure S3 — Similarity in physico-chemical properties within coevolved residues. Correlation coefficients (X axis) were calculated between two coevolved sites utilizing matrices (values normalized from 0 to 1) of 13 non redundant physico-chemical properties (such as hydrophobicity, polarity, charge etc) obtained from the UMBC AAIndex database. Non-coevolved pairs were selected by randomly picking two sites from a pool of non-coevolving sites within each protein family. Histogram corresponding to the coevolved sites is shifted toward larger positive values compared to the histogram of correlation coefficients calculated for randomly selected non-coevolved sites (p-value<10−4) indicating that coevolved sites have more similar physico-chemical properties compared non-coevolved sites. (0.10 MB DOC) [file pone.0008591.s003.doc]

Supporting Information File 5:

Authors: Saikat Chakrabarti and Anna R. Panchenko

**Figure S4**


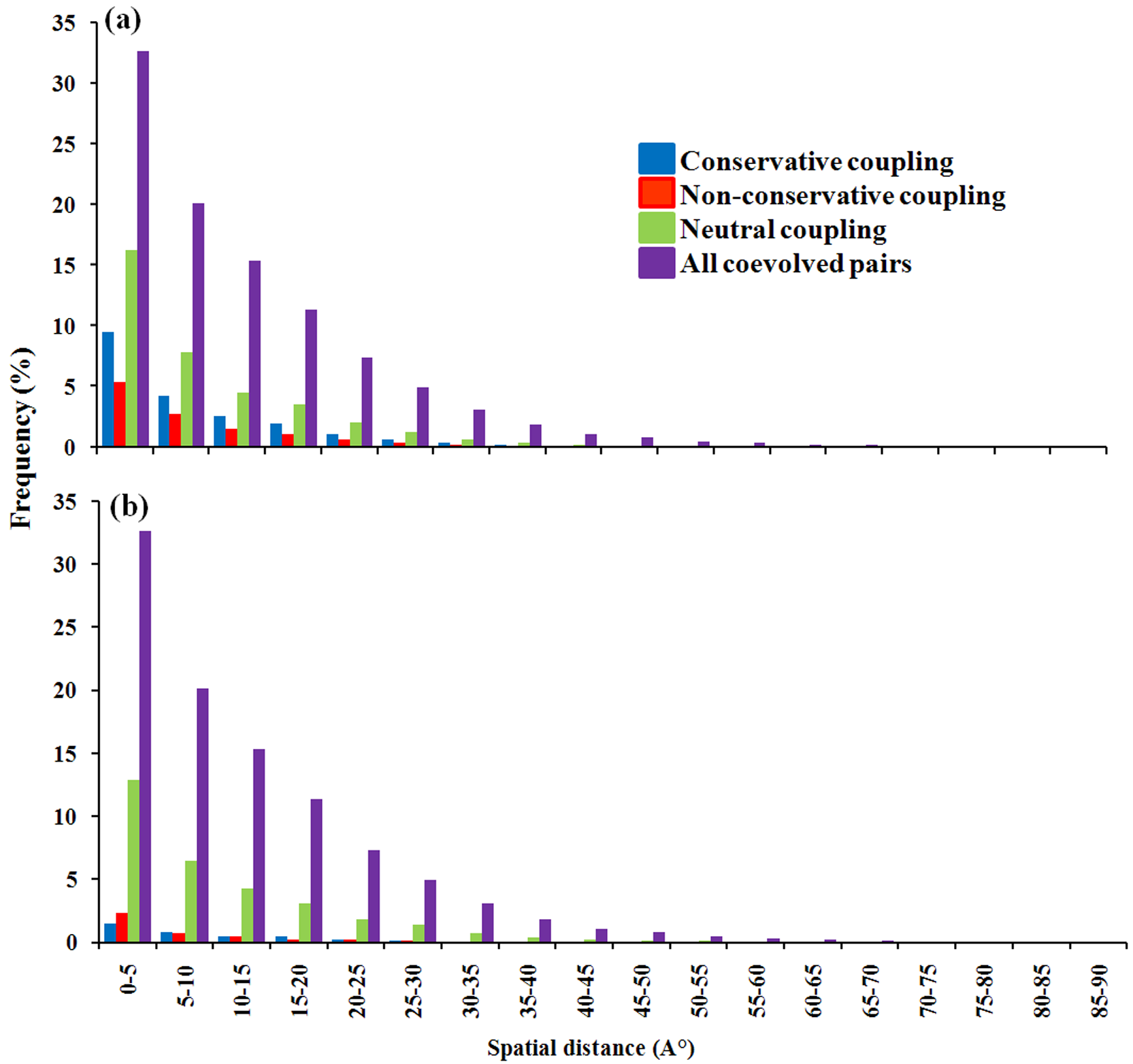

Supplement: Figure S4 — Conservative and non-conservative coevolved residue pairs. Residue pairs were categorized according to volume (a) and charge (b) of amino acids. Covariations between residue pairs differing not more than volume of a methyl group (∼30Å) are termed as ‘conservative coupling’ where residue pairs with volume difference of more than one or two methyl groups are categorized as neutral coupling. Larger volume deviations are marked as ‘non-conservative coupling’ (please see Table S5 for details). Similarly, pairing between two oppositely charged residues is termed as ‘non-conservative’ while charge a ‘conservative coupling’ is constituted by two similarly charged residues. Coevolved residue pairs where one residue is charged while the other is not are termed as ‘neutral coupling’. Frequencies (normalized by the number of all coevolved residue pairs) of conservative, non-conservative, and neutral residue pairs were plotted with respect to the spatial distances (X axis) between the coevolved residue pairs. (0.26 MB DOC) [file pone.0008591.s004.doc]

Supporting Information File 6:

Authors: Saikat Chakrabarti and Anna R. Panchenko

**Figure S5.**

**
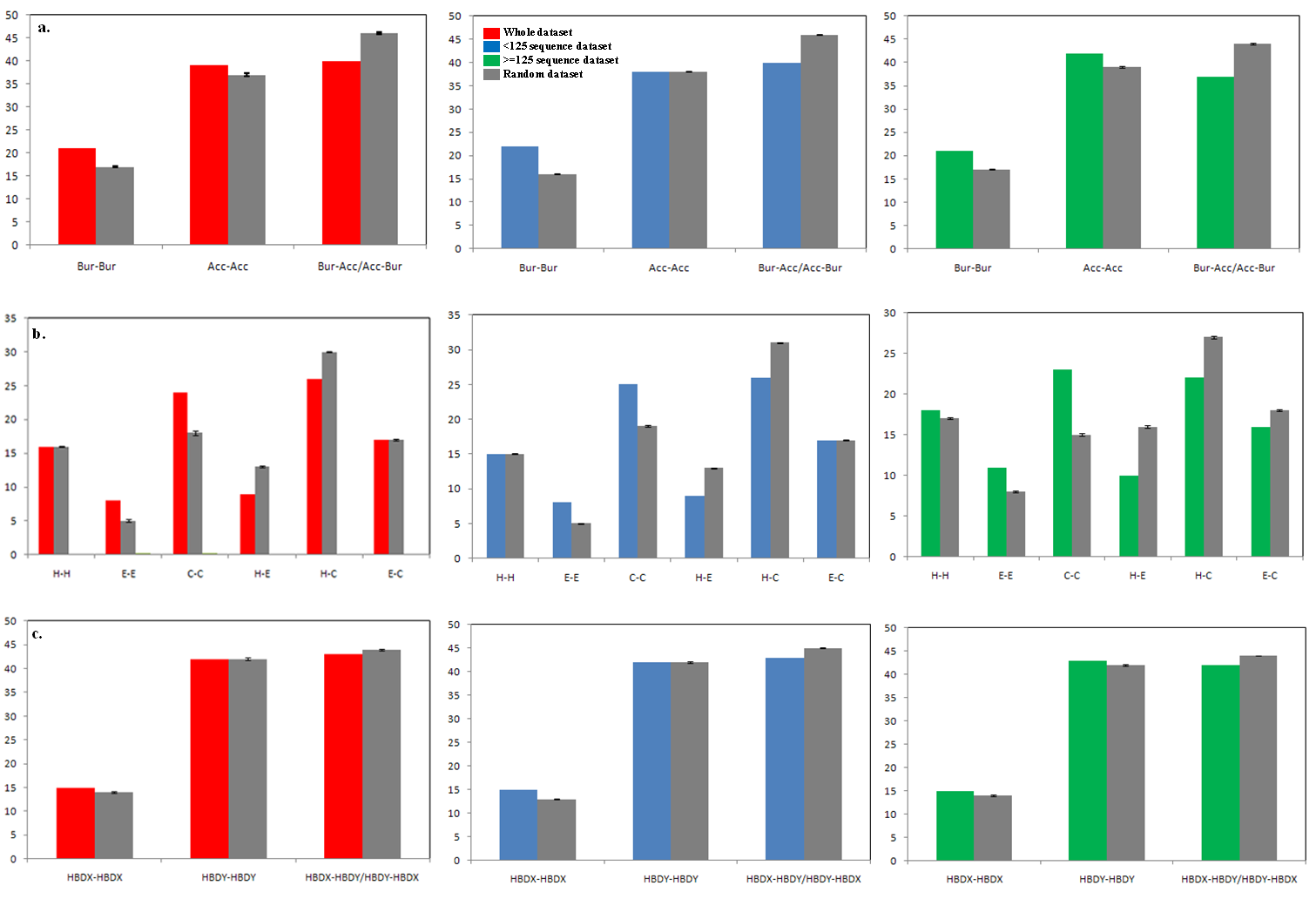
**

Supplement: Figure S5 — Structural properties such as solvent accessibility (panel a), type of secondary structures (panel b) and hydrogen bonds (panel c) for the coevolved sites were compared with that of randomly selected non-coevolved site pairs. (0.18 MB DOC) [file pone.0008591.s005.doc]
