# Supplementary material for: Structural and Functional Roles of Coevolved Sites in Proteins
Source: PLoS One. 2010 Jan 6;5(1):e8591. doi: 10.1371/journal.pone.0008591 (PMC2797611; doi:10.1371/journal.pone.0008591)
Supplement: Table S1 — Mean distances for randomly selected non-coevolved site pairs are provided within parenthesis. Significance of the test was determined with respect to the mean distances of randomly selected non-coevolved pairs (1000 randomization cycles). (0.03 MB DOC) [file pone.0008591.s006.doc]

Supporting Information File 2:

Authors: Saikat Chakrabarti and Anna R. Panchenko

**Table S1:** Mean distances of coevolved sites.

| **Alignments** | **Mean Distance (Å)** | **Number of coevolved pairs** | **Mean separation (residues)** | **Significance** |
| --- | --- | --- | --- | --- |
| Whole dataset (803 alignments) | 12.8 (24.7) | 39527 | 65.9 | <1E-3 |
| <125Seq dataset (622 alignments) | 13.8 (25.9) | 31849 | 71.0 | <1E-3 |
| >=125Seq dataset (181 alignments) | 8.7 (21.3) | 7678 | 44.8 | <1E-3 |
