# Supplementary material for: Structural and Functional Roles of Coevolved Sites in Proteins
Source: PLoS One. 2010 Jan 6;5(1):e8591. doi: 10.1371/journal.pone.0008591 (PMC2797611; doi:10.1371/journal.pone.0008591)
Supplement: Table S4 — (0.03 MB DOC) [file pone.0008591.s009.doc]

Supporting Information File 9:

Authors: Saikat Chakrabarti and Anna R. Panchenko

**Table S4: List of physico-chemical properties used to test similarity between coevolved sites**

**1. Volume**

**2. Polarity**

**3. Hydrophobicity index**

**4. Hydration potential**

**5. Occurrence frequency**

**6. Absolute entropy**

**7. Relative mutability**

**8. Net charge**

**9. Isoelectric point**

**10. pk-a Rcooh**

**11. Normalized flexibility parameters**

**12. Side chain orientation preference**

**13. 14Å contact number**
