# Supplementary material for: Structural and Functional Roles of Coevolved Sites in Proteins
Source: PLoS One. 2010 Jan 6;5(1):e8591. doi: 10.1371/journal.pone.0008591 (PMC2797611; doi:10.1371/journal.pone.0008591)
Supplement: Table S5 — (0.03 MB DOC) [file pone.0008591.s010.doc]

Supporting Information File 10:

Authors: Saikat Chakrabarti and Anna R. Panchenko

**Table S5: List of residue couplings.**

| **Property of amino acids** | **Type of coupling** | **Amino acid coupling** |
| --- | --- | --- |
| Volume | Conservative | G<->A, G<->S, A<->S, P<->V, P<->N, P<->T, P<->C, V<->N, V<->T, V<->C, N<->T, N<->C, T<->C, L<->I, L<->M, L<->Q, L<->H, I<->M, I<->Q, I<->H, M<->Q, M<->H, Q<->H, F<->Y |
|  | Non-conservative | G<->L, G<->I, G<->M, G<->Q, G<->H, G<->F, G<->Y, G<->W, A<->F, A<->Y, A<->W, S<->F, S<->Y, S<->W, P<->F, P<->Y, P<->W, V<->F, V<->Y, V<->W, N<->F, N<->Y, N<->W, T <->F, T<->Y,T<->W, C<->F, C<->Y, C<->W |
|  | Neutral | All other hetero amino acids pairs excluding the charged amino acids. |
| Charge | Conservative | D<->E, R<->K |
|  | Non-conservative | D<->R, D<->K, E<->R, E<->K |
|  | Neutral | All other hetero amino acids pairs where one residue of the pair is charged. |
